# Photosynthetic Physiological Response of Radix Isatidis (*Isatis indigotica* Fort.) Seedlings to Nicosulfuron

**DOI:** 10.1371/journal.pone.0105310

**Published:** 2014-08-28

**Authors:** Xiangyang Yuan, Liguang Zhang, Na Ning, Yinyuan Wen, Shuqi Dong, Meiqiang Yin, Meijun Guo, Binqiang Wang, Lei Feng, Pingyi Guo

**Affiliations:** 1 Laboratory of Crop Chemical Regulation and Chemical Weed Control, Agronomy College, Shanxi Agricultural University, Taigu, the People's Republic of China; 2 Institute of Crop Sciences, Shanxi Academy of Agricultural Sciences, Taiyuan, the People's Republic of China; University of Vigo, Spain

## Abstract

Radix Isatidis (Isatis indigotica Fort.) is one of the most important traditional Chinese medicine plants. However, there is no suitable herbicide used for weed control in Radix Isatidis field during postemergence stage. To explore the safety of sulfonylurea herbicide nicosulfuron on Radix Isatidis (Isatis indigotica Fort.) seedlings and the photosynthetic physiological response of the plant to the herbicide, biological mass, leaf area, photosynthetic pigment content, photosynthetic rate, chlorophyll fluorescence characteristics, and P_700_ parameters of Radix Isatidis seedlings were analyzed 10 d after nicosulfuron treatment at 5th leaf stage in this greenhouse research. The results showed that biological mass, total chlorophyll, chlorophyll a, and carotenoids content, photosynthetic rate, stomatal conductance, PS II maximum quantum yield, PS II effective quantum yield, PS II electron transport rate, photochemical quenching, maximal P_700_ change, photochemical quantum yield of PS I, and PS I electron transport rate decreased with increasing herbicide concentrations, whereas initial fluorescence, quantum yield of non-regulated energy dissipation in PS II and quantum yield of non-photochemical energy dissipation due to acceptor side limitation in PS I increased. It suggests that nicosulfuron ≥1 mg L−1 causes the damage of chloroplast, PS II and PS I structure. Electron transport limitations in PS I receptor side, and blocked dark reaction process may be the main cause of the significantly inhibited growth and decreased photosynthetic rate of Radix Isatidis seedlings.

## Introduction

As one of the most important traditional Chinese medicine plants, Radix Isatidis (*Isatis indigotica* Fort.), one kind of cruciferous plants, is extensively cultivated in China. However, weeds seriously limit its yield and quality. Compared with conventional manual control of weeds, chemical control is more effective, yet there is no suitable herbicide used for weed control in Radix Isatidis field during postemergence stage [1]. Nicosulfuron (2-((4,6-dimethoxypyrimidin-2-ylcarbamoyl)sulfamoyl)-N, N-dimethylnicotinamide) belongs to the acetyl lactic acid synthase (ALS) inhibitor, and can effectively control many perennial and annual grasses as well as certain broadleaf weeds [2], [3]. Is nicosulfuron safe to Radix Isatidis seedlings or not?

It is reported that nicosulfuron can effectively control large crabgrass (*Digitaria sanguinalis*) cornfield, goosegrass (*Eleusine indica*), spiny amaranth (*Amaranthus spinosus* L), amaranthus blitum (*Amaranthus ascendens* Loisel.), speargrass (*Imperata cylindrica*), and purslane (*Portulaca oleracea*) in maize field [4]. However, there are significant differences during the sensitivity of maize varieties to nicosulfuron [5], and sweet corn is much more sensitive to nicosulfuron than the others [6]. The leaves of sensitive maize variety may show symptoms of chlorosis and shrinking 7 d after applying nicosulfuron [7]. The weeding efficiency of nicosulfuron herbicide is related to weed species, leaf age and how long it is applied after rain. [8]. Meanwhile, the resistance of plant to nicosulfuron depends on its metabolic rate, sensitivity to ALS, and nicosulfuron dosage [9]–[11].

However, there is few paper reporting the application of post-emergence herbicide controlling many grasses and broadleaf weeds in Radix Isatidis fields [1]. It is shown that rational application of trifluralin, pendimethalin and glyphosate at pre-emergence stage can control weeds effectively in Radix Isatidis fields [12], [13]. It is also reported that clethodim and quizalofop-p-ethyl at post-emergence stage can be used for grassy weed control in Radix Isatidis fields [12], [14].

Primary mode of action of the ALS-inhibiting herbicides that interfere with the activity of ALS enzyme seems no longer in doubt [15]. But, secondary effects of ALS inhibition, such as decreased photosynthesis, disturbed respiration, and synthesis of branched chain amino acids, etc., need to be investigated, which have also been implicated in the mechanism of plant death. Detection of chlorophyll fluorescence dynamics is a rapid and non-invasive probe of researching plant photosynthetic functions, which has been widely applied to study the effects of herbicides in plants [1], [16]–[19]. Therefore, the objectives of this research were to (1) assess the possibility of application of nicosulfuron in Radix Isatidis field, and (2) understand the related photosynthetic physiological mechanism.

## Materials and Methods

### Materials and experiment design

Radix Isatidis (*Isatis indigotica* Fort.) seeds were supplied by Anguo Lixin Medicinal Materials Co., Ltd., Hebei province, China. Nicosulfuron (40%, OF) was provided by Xianda Chemical Co., Ltd., Shandong province, China.

This study was conducted in a greenhouse at Shanxi Agricultural University, China. The experiment was designed as a randomized complete block design with three replications and each replicate containing three pots. Fifteen Radix Isatidis seeds were grown equidistantly in 23-cm diam containers filled with a 1∶2 mixture of sand and loam soil with 57.2 g kg^−1^ of organic matter, 0.92 g kg^−1^ of total nitrogen, 14.37 mg kg^−1^ of available phosphorus, and 114.3 mg kg^−1^ of rapidly-available potassium. The seeds were covered with 1 cm of 1∶2 sand/soil mixtures and each pot was carefully watered. Seedlings were grown under greenhouse conditions of 24/16±3°C day/night temperatures and were thinned to three plants per container at three-leaf stage.

Radix Isatidis seedlings were treated at five-leaf stage. These plants were treated with 0, 0.5, 1, 2, and 4 times the labeled use concentrations of nicosulfuron in corn. The herbicide product contained 40% nicosulfuron, large amounts of water and a small amount of oil adjuvants, and the recommended effective concentrations were 1 mg L^−1^ nicosulfuron. Herbicides were applied with a laboratory pot-sprayer equipped with a nozzle, calibrated to deliver 450 L ha^−1^. Agronomic characters and photosynthetic physiology parameters of Radix Isatidis seedling were determined every 10 d after herbicide treatment. Except the control, seedlings treated with nicosulfuron wilted or died 20 d after herbicide treatment, so the data 10 d after herbicide treatment was determined.

### Measurements

The third fully expanded leaf of Radix Isatidis seedlings were sampled for the following tests. Leaf area was determined using a laser leaf area meter CI-203 and the CI-203CA conveyor attachment (United States CID Inc.).

Photosynthetic gas exchange was analyzed with a GFS-3000 optical instrument (Germany WALZ company) which can control photosynthesis by means of light intensity, leaf temperature, air flow rate and CO_2_ concentration in the cuvette. Photosynthetic rate (*P*_n_), transpiration rate (*T*_r_), stomatal conductance (*G*_s_) and intercellular CO_2_ concentrations (*C*_i_) were measured simultaneously with the light intensity at (800±0.4 µmol·m^−2^·s^−1^) and CO_2_ concentration (379±0.4 µmol·mol^−1^). Air flow rate was set at 750 µmol s^−1^ and air temperature (20.9±0.4°C) was also recorded automatically by the instrument. Stomatal limitation value (*L*_s_) = 1–*C*_i_/*C*_a_ (*C*_a_ is the atmospheric CO_2_ concentration). Non-stomatal limitation value was calculated by *C*_i_/*G*_s_ [20]. For the measurement of photosynthetic pigments, leaves were extracted from leaf discs with 80% (v/v) acetone and assayed spectrophotometrically using extinction coefficients according to Porra et al. [21].

Chlorophyll fluorescence and *P*_700_ parameters were measured simultaneously by Dual-PAM-100 measurement system (Germany WALZ company), using the automated ‘‘Induction Curve’’ routine provided by the Dual PAM software [22]. Prior to measurements, treated plants were placed in darkroom for 30 min, and fluorescence induced curve (Slow Kinetics) was determined in ‘‘Fluo+*P*_700_ mode’’. Then, the kinetics of chlorophyll fluorescence induction and *P*_700_ oxidation were recorded simultaneously by the instrument. Firstly, the initial fluorescence (*F*_o_) was established and subsequently the maximum fluorescence (*F*_m_) was determined by the ‘‘Saturation Pulse’’ method. Secondly, the maximal *P*_700_ change (*P*_m_) was determined by application of a saturation pulse (SP) after far-red pre-illumination. Thirdly, actinic illumination was started and SP was given every 20 s, with the same pulses serving for fluorescence and *P*_700_ analysis.

PS II maximum quantum yield (*F*_v_/*F*_m_) was evaluated as *F*_v_/*F*_m_ = (*F*_m_–*F*_o_)/*F*_m_. Other PS II energy dissipation parameters were estimated by the Dual PAM software. *q*_P_ = (*F*_m_ *’*–*F*)/(*F*_m_ *’*–*F*_o_ *’*) was used as indicator to reflect a ratio of light energy absorbed in PS II being used to photochemical electron transport. Apparent electron transfer efficiency in PS II in light was calculated according to ETR(II) = PAR×0.84×0.5×Y(II), and was used to measure electron transfer of carbon fixation resulted from photochemical reactions. Three complementary quantum yields of energy conversion in PS II were calculated: PS II effective quantum yield (Y(II)) was evaluated as (Y(II)) = (*F*_m_ *’*–*F*)/*F*_m_ *’*, the yield of non-photochemical losses via non-regulated pathways of PS II as Y(NO) = 1/(NPQ+1+*q*_L_·(*F*_m_/*F*_o_–1))), NPQ = *F*_m_/*F*_m_ *’*–1, *q*_L_ = *q*_P_·*F*_o_ *’/F*, where quantum yield of regulated energy dissipation in PS II as Y(NPQ) = 1–Y(II)–Y(NO) [23].

*P*_700_ oxidation was monitored by absorbance changes in the near-infrared (830–875 nm) [24]. The maximal *P*_700_ signal observed upon full oxidation was denoted by Pm. Y(NA), the quantum yield of non-photochemical energy dissipation due to acceptor-side limitation, was calculated according to: Y(NA) = (*P*_m_ *’–P*_m_)/*P*_m_ *’*. Photochemical quantum yield of PSI as Y(I) was estimated according to Y(I) = (*P*_m_’–*P*)/*P*_m_; Quantum yield of non-photochemical energy dissipation due to donor side limitation in PS I as Y(ND) was calculated by Y(ND) = (*P*–*P*_o_)/*P*_m_. The total value of three quantum yields was one: Y(I)+Y(ND)+Y(NA) = 1. The electron transfer efficiency of PS I as ETR (I) was provided by the Dual PAM software.

### Statistical analysis

Microsoft Office Excel 2003 and Statistics Analysis System 8.0 were used in statistical analysis of the data. Mean values were compared by a one-way analysis of variance (ANOVA), and Duncan’s test was used to determine the significant differences among the treatments. We used P = 0.05 as the statistical significance threshold.

## Results

### Effect of nicosulfuron on agronomic characteristics of Radix Isatidis seedlings

The effects of nicosulfuron on agronomic characteristics of Radix Isatidis seedlings are shown in Table 1. Fresh weight of Radix Isatidis seedlings decreased with the increasing concentrations of nicosulfuron. Leaf area was significantly decreased by nicosulfuron at 4 mg L^−1^, whereas this was not affected by other treatment. The whole plant fresh weight and shoot fresh weight showed the similar trend, and differences between the treatment of nicosulfuron at 0.5 mg L^−1^ and the control were significant. Shoot fresh weight declined by 41.64%, 52.90%, 53.58% and 59.04%, respectively, from 0.5 to 4 mg L^−1^ of nicosulfuron. The reduction in root fresh weight was not significant by nicosulfuron, suggesting that the suppression of nicosulfuron on the aboveground parts of Radix Isatidis was greater than root.

**Table 1:** Effect of different concentrations of nicosulfuron on agronomic traits of radix isatidis seedlings.

Nicosulfuron (mg L^−1^)	*LA* (cm^2^)	*WPFW* (g)	*SFW* (g)	*RFW* (g)
0	10.54±0.24^a^	3.18±0.04^a^	2.93±0.05^a^	0.25±0.05^a^
0.5	9.93±0.75^a^	1.94±0.18^b^	1.71±0.16^b^	0.23±0.03^a^
1	11.11±0.37^a^	1.58±0.10^bc^	1.38±0.04^bc^	0.20±0.04^a^
2	9.54±0.24^a^	1.52±0.14^bc^	1.36±0.27^bc^	0.17±0.02^a^
4	7.37±0.71^b^	1.36±0.29^c^	1.20±0.18^c^	0.16±0.01^a^

Note: The data in the table is mean ± SD. The different letters in the same column indicate significantly different at P<0.05 level by Duncan’s new multiple range test. *LA*, *WPFW*, *SFW* and *RFW* represent leaf area, the whole plant fresh weight, shoot fresh weight, and root fresh weight, respectively.

### Effect of nicosulfuron on photosynthetic pigment contents in leaves of Radix Isatidis seedlings

As shown in Table 2, each nicosulfuron treatment caused different degrees of decline in photosynthetic pigment contents in leaves of Radix Isatidis seedlings. It seems that 1 mg L^−1^ of nicosulfuron inhibited *Chl* by 33.57%, *Chl* a by 37.04% and *Car* by 47.37, and the differences between the treatment and the control were significant. However, *Chl* b was not significantly decreased until the herbicide concentrations reached up to 2 mg L^−1^, and it was inhibited by 37.14%. Although nicosulfuron declined chlorophyll a/b, there was no significant effect.

**Table 2:** Effect of different concentrations of nicosulfuron on photosynthetic pigment content in leaves of Radix isatidis seedling.

Nicosulfuron (mg L^−1^)	*Chl* (mg g^−1^)	*Chl* a (mg g^−1^)	*Chl* b (mg g^−1^)	*Car* (mg g^−1^)	*Chl* a/b
0	1.43±0.10^a^	1.08±0.08^a^	0.35±0.01^a^	0.19±0.01^a^	3.12±0.11^a^
0.5	1.00±0.03^b^	0.75±0.02^b^	0.25±0.05^ab^	0.13±0.02^b^	2.98±0.68^a^
1	0.95±0.05^bc^	0.68±0.001^bc^	0.27±0.04^abc^	0.10±0.01^b^	2.49±0.35^a^
2	0.82±0.07^c^	0.60±0.09^c^	0.22±0.02^bc^	0.12±0.01^bc^	2.76±0.61^a^
4	0.57±0.11^d^	0.40±0.09^d^	0.17±0.02^d^	0.08±0.02^c^	2.34±0.30^a^

Note: The data in the table is mean ± SD. The different letters in the same column indicate significantly different at P<0.05 level by Duncan’s new multiple range test. *Chl*, *Chl* a, *Chl* b, *Car* and *Chl* a/b represent chlorophyll, chlorophyll a, chlorophyll b, carotenoid, and chlorophyll a/b, respectively.

### Effect of nicosulfuron on photosynthetic characteristics in leaves of Radix Isatidis seedlings

Nicosulfuron decreased *P*_n_ and *G*_s_ in leaves of Radix Isatidis seedling significantly. As concentration of nicosulfuron increases, inhibition of *P*_n_ changed by 58.21%, 70.02%, 70.69%, and 78.89%, respectively (Table 3). *G*_s_ inhibition also increases as concentration of the herbicide increases by 86.03%, 91.10%, 91.21%, and 96.49%, respectively when nicosulfuron varies from 0.5 to 4 mg L^−1^ (Table 3). However, the results of *P*_n_ and *G*_s_ revealed no significant differences (P>0.05) from 1 to 4 mg L^−1^ of nicosulfuron. *C*_i_ increased first then declined with increasing of nicosulfuron concentrations, peaked at 0.5 mg L^−1^, and was the lowest at 4 mg L^−1^. Compared to *C*_i_, the change trend of *L*_s_ was just the opposite. *L*_s_ was the least at 4 mg L^−1^, and the largest at 1 mg L^−1^. For *C*_i_/*G*_s_, the value for treated plants was higher than the control in different degrees.

**Table 3:** Effect of different concentrations of nicosulfuron on photosynthetic characteristics in leaves of Radix isatidis seedling.

Nicosulfuron(mg L^−1^)	*P*_n_ (µmolCO_2_·m^−2^·s^−1^)	*C*_i_(µmol·mol^−1^)	*G*_s_(mmolH_2_O·m^−2^·s^−1^)	*L*_s_	*C*_i_ */G*_s_
0	11.94±1.857^a^	296.30±10.62^a^	264.57±76.31^a^	0.21±0.03^a^	1.16±0.29^c^
0.5	4.99±0.23^b^	336.31±13.18^ab^	36.96±0.24^b^	0.11±0.04^ab^	9.10±0.30^bc^
1	3.58±1.26^b^	309.20±70.20^ab^	23.54±13.04^b^	0.05±0.01^b^	19.98±7.05^ab^
2	3.50±0.60^b^	235.73±63.10^b^	23.25±14.89^b^	0.38±0.17^b^	13.85±1.58^ab^
4	2.52±1.57^b^	115.10±10.38^c^	9.29±17.92^b^	0.70±0.01^b^	21.60±2.32^a^

Note: The data in the table is mean±SD. The different letters in the same column indicate significantly different at P<0.05 level by Duncan’s new multiple range test. *P*_n_, *C*_i_, *G*_s_, *L*_s_ and *C*_i_ */G*_s_ represent photosynthetic rate, intercellular CO_2_ concentrations, stomatal conductance, stomatal limitation value, and non-stomatal limitation value, respectively.

### Effect of nicosulfuron on chlorophyll fluorescence parameters in leaves of Radix Isatidis seedlings

**Figure 1:**
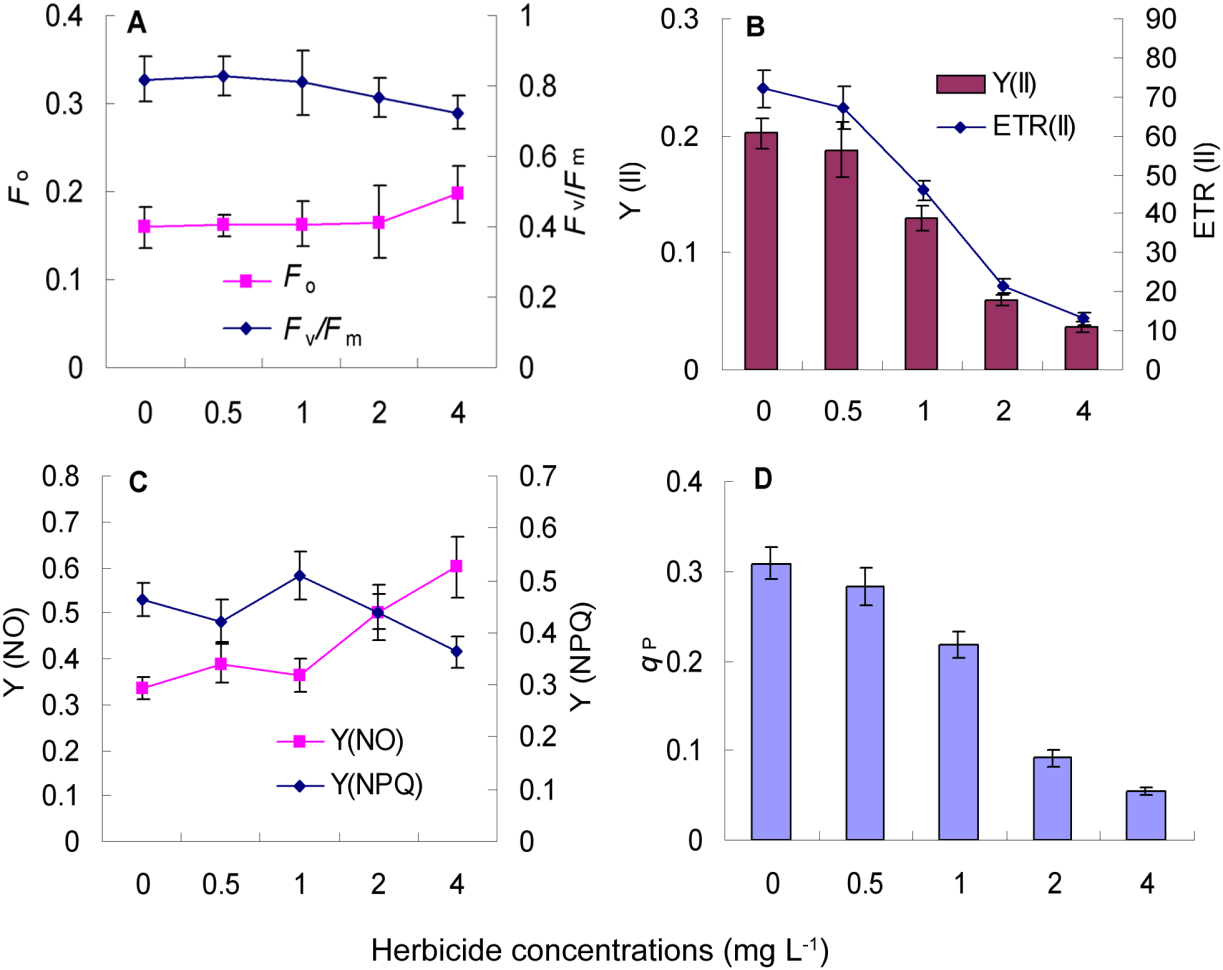
Effect of nicosulfuron on chlorophyll fluorescence parameters in leaves of Radix Isatidis seedlings. Values represent the means and vertical bars indicate the standard deviation of three separate experiments. *F*_o_, *F*_v_/*F*_m_, Y(II), ETR (II), Y(NO), Y(NPQ) and *q*_P_ represent initial fluorescence, PS II maximum quantum yield, PS II electron transport rate, PS II effective quantum yield, quantum yield of non-regulated energy dissipation in PS II, quantum yield of regulated energy dissipation in PS II, and photochemical quenching, respectively. The abscissa in the figure represents the concentration of nicosulfuron and the unit is “mg L^−1^”.

As shown in Fig. 1-A, *F*_o_ increased with the increase in nicosulfuron concentration. *F*_o_ was a bit higher than the control for 2 mg L^−1^ treatment, but significantly higher for 4 mg L^−1^ treatment. *F*_v_/*F*_m_ was slightly higher than the control for 0.5 mg L^−1^ concentration, and then decreased with the increase concentrations of nicosulfuron.

Changes of Y(II), ETR and *q*_P_ were consistent, and declined in a concentration-dependent manner. Compared with the control, Y(II) was reduced by 7.01%, 36.07%, 70.57% and 81.95%, ETR declined by 7.10%, 36.14%, 70.44% and 81.91%, and *q*_P_ decreased by 8.64%, 29.54%, 70.19% and 82.51%, respectively (Fig. 1-B, 1-D).

Y(NO) showed a fluctuant increase as nicosulfuron concentration increases, slightly higher than the control for 0.5 mg L^−1^ and 1 mg L^−1^ concentration, and significantly (49.70%) higher for 2 mg L^−1^. Compared to Y(NO), the trend of Y(NPQ) was the opposite (Fig. 1-C). It showed that the degree of the relative excess light damage induced by nicosulfuron in Radix Isatidis leaf was more severe with increasing nicosulfuron concentration.

### Effect of nicosulfuron on *P*_700_ parameters in leaves of Radix Isatidis seedlings

**Figure 2:**
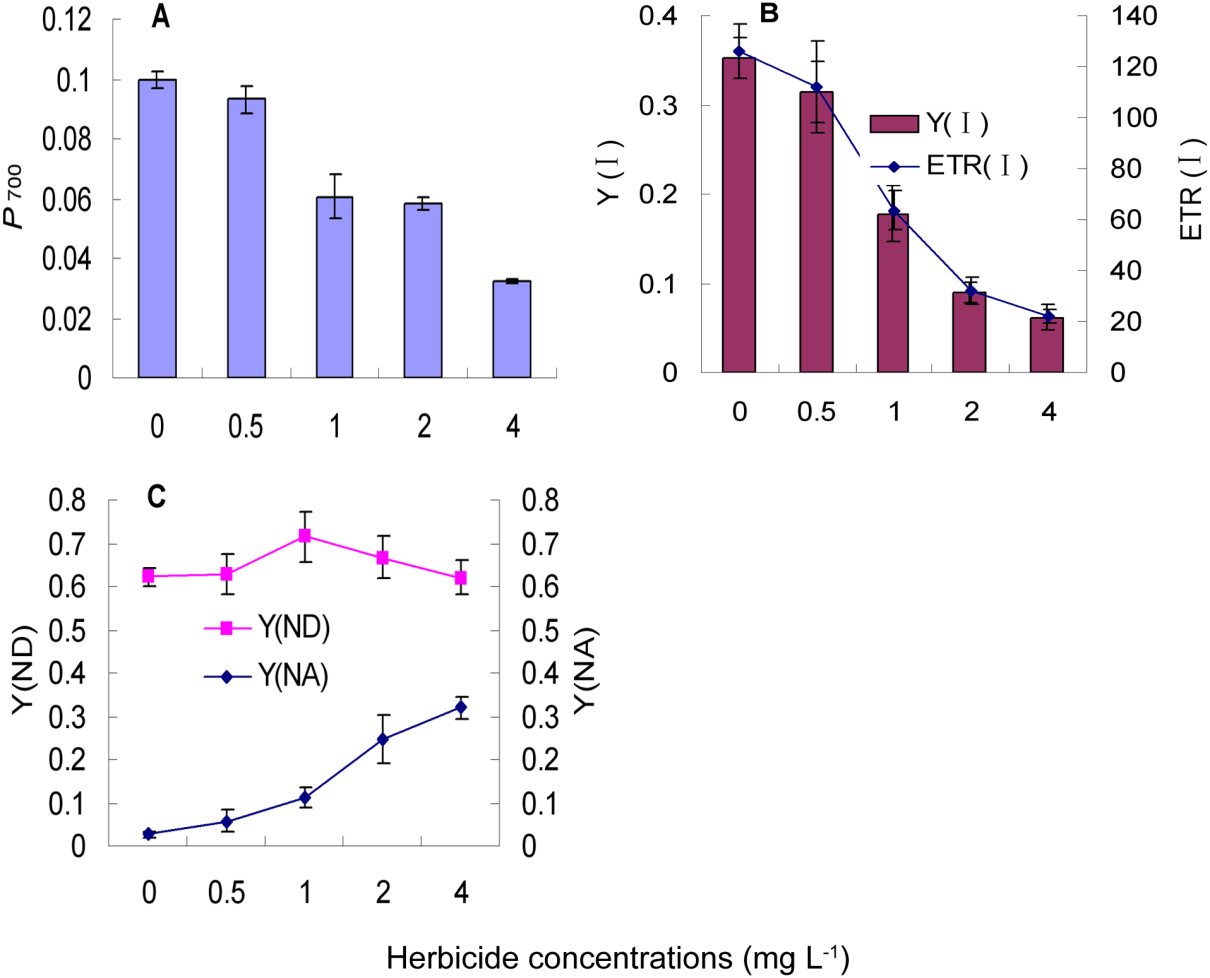
Effect of nicosulfuron on P700 parameters in leaves of Radix Isatidis seedlings. Values represent the means and vertical bars indicate the standard deviation of three separate experiments. *P*_m_, Y(I), ETR (I), Y(ND) and Y(NA) represent maximal *P*_700_ change, photochemical quantum yield of PS I, PS I electron transport rate, quantum yield of non-photochemical energy dissipation due to donor side limitation in PS I, and quantum yield of non-photochemical energy dissipation due to acceptor side limitation in PS I. The abscissa in the figure represents the concentration of nicosulfuron and the unit is “mg L^−1^”.

Increasing nicosulfuron concentration induced a decline in *P*_m_, ETR (I), and PS I. Nicosulfuron treatment at 1 mg L^−1^ had dramatically influence on *P*_m_, while the differences between 1 mg L^−1^ and 2 mg L^−1^ were not significant (Fig. 2-A). As shown in Fig. 2-B, the trend of ETR (I) and PS I was consistent, and the values of them at 2 mg L^−1^ were much smaller than 1 mg L^−1^ (Fig. 2-B). In Fig. 2-C, nicosulfuron induced an increase of Y(NA), while Y(ND) increased first and then decreased with the increasing of nicosulfuron concentration, and the peak was at 1 mg L^−1^ (Fig. 2-C).

## Discussion

The safety of herbicides on plants may be represented through agronomic traits and physiological indexes [1], [25], [26]. In this study, nicosulfuron at the recommended usage (1 mg L^−1^) was not safe to Radix Isatidis seedlings, which was reflected by reduced biomass. It was coincident with Yuan et al. [1], mesosulfuron-iodosulfuron was unsafe to Radix Isatidis seedlings and decreased the leaf area and fresh weight significantly. Meanwhile, nicosulfuron was unsafe to Radix Isatidis seedlings being reflected by reduced chlorophyll content and *P*_n_.

Is the decline in *P*_n_ resulted by stomatal factors or non-stomatal factors? In our current research, *P*_n_ and *G*_s_ at 0.5 mg L^−1^ were significant lower than the control, however, *C*_i_ and *C*_i_/*G*_s_ were a little higher than the control. It may suggest that the decline in *P*_n_ is mainly caused by stomatal limitation. *P*_n_, *G*_s_ and *C*_i_ treated by nicosulfuron ≥2 mg L^−1^ were significantly lower than the control, and *L*_s_ and *C*_i_/*G*_s_ were significant higher than the control, showing that both stomatal and non-stomatal factors may limit the photosynthesis. Nicosulfuron at 0.5 mg L^−1^ decreased *Chl* a and *Car* content significantly. It may suggest that nicosulfuron destructs the chloroplast structure of Radix Isatidis leaf, reduces the thylakoid stacking level [27], increases the risk of photo-oxidation damage, reduces the light absorption, transmission, distribution between PS II and PSI [28], and affects the synthesis of ATP and NADPH.

Chloroplasts of photosynthetic apparatus, PS II and PS I in thylakoid membranes are the most sensitive parts to environmental changes. Reversible inactivation or damage of PS II reaction center can cause increase in *F*_o_ [29]. Paraquat and norflurazon at 100 µg L^−1^ significantly reduced *F*_v_/*F*_m_, Y(II) and *q*_P_ in leaves of Lemna minor, but enhanced NPQ markedly [30]. Acetochlor and fluoroglycofen decreased the photochemical efficiency of photosystem II (Y(II)) in the light and increased non-photochemical quenching (NPQ) [17]. Previous studies [19] have shown that Sigma Broad causes damage to PS II complex, block photosynthetic electron transfer, reduce *F*_v_/*F*_m_, Y(II) and *q*_P_ significantly, and lead to increase in initial fluorescence, quantum yield of non-regulated energy dissipation in PS II in Radix Isatidis seedlings. Similar to the previous, *F*_o_ and Y(NO) increased, whereas *F*_v_/*F*_m_, Y(II), ETR(II), *q*_P_ and Y(NPQ) decreased in leaves treated by nicosulfuron in this study. It may suggest that nicosulfuron causes excess excitation energy accumulation in PS II reaction center, the higher reduction state of *Q*_A_, net loss of D_1_ protein, reversible deactivation or destruction in PS II reaction centers [31], opening percentage to decrease, harmful effect on photosynthetic oxygen-evolving complex, electron transport efficiency to decline, ATP and NADPH to reduce in Radix Isatidis leaves.

Typical feature of PS I photoinhibition is the decline in maximum oxidation-reduction ability of PS I [32]. In this research, *P*_m_, Y(I) and ETR (I) declined with the increase of nicosulfuron usage, and which at 1 mg L^−1^ were significantly lower than the control. It may suggest that herbicide suppressed the activity of PS I in Radix Isatidis leaves, and electron transfer was blocked at its receptor side. Y(ND) reflects the state of electron donor in PS I, and it is affected by the transmembrane proton gradient and PS II damage degree. Y(NA) reflects the state of electron acceptor in PS I, and it is affected by dark adaptation and CO_2_ fixation damage level. Y(NA) >0.5 mg L^−1^ increased significantly, showing that nicosulfuron aggravated the injury of PS II in Radix Isatidis leaves, declined transmembrane proton gradient, blocked dark reaction process, and reduced the fixed amount of CO_2_.

In conclusion, recommended usage of nicosulfuron for maize is not safe to Radix Isatidis seedlings. It causes the damage of chloroplast, PS II and PS I structure. Electron transport limitations in PS I receptor side, blocked dark reaction process may be the main cause of the significantly inhibited growth and decreased photosynthetic rate of Radix Isatidis seedlings. Effect of nicosulfuron on the activities of key enzymes in the Calvin cycle of Radix Isatidis seedlings will be researched in the later experiment.
